# Non-invasive genetics outperforms morphological methods in faecal dietary analysis, revealing wild boar as a considerable conservation concern for ground-nesting birds

**DOI:** 10.1371/journal.pone.0179463

**Published:** 2017-06-08

**Authors:** Ragne Oja, Egle Soe, Harri Valdmann, Urmas Saarma

**Affiliations:** Department of Zoology, Institute of Ecology and Earth Sciences, University of Tartu, Tartu, Estonia; Universita degli Studi di Sassari, ITALY

## Abstract

Capercaillie (*Tetrao urogallus*) and other grouse species represent conservation concerns across Europe due to their negative abundance trends. In addition to habitat deterioration, predation is considered a major factor contributing to population declines. While the role of generalist predators on grouse predation is relatively well known, the impact of the omnivorous wild boar has remained elusive. We hypothesize that wild boar is an important predator of ground-nesting birds, but has been neglected as a bird predator because traditional morphological methods underestimate the proportion of birds in wild boar diet. To distinguish between different mammalian predator species, as well as different grouse prey species, we developed a molecular method based on the analysis of mitochondrial DNA that allows accurate species identification. We collected 109 wild boar faeces at protected capercaillie leks and surrounding areas and analysed bird consumption using genetic methods and classical morphological examination. Genetic analysis revealed that the proportion of birds in wild boar faeces was significantly higher (17.3%; 4.5×) than indicated by morphological examination (3.8%). Moreover, the genetic method allowed considerably more precise taxonomic identification of consumed birds compared to morphological analysis. Our results demonstrate: (i) the value of using genetic approaches in faecal dietary analysis due to their higher sensitivity, and (ii) that wild boar is an important predator of ground-nesting birds, deserving serious consideration in conservation planning for capercaillie and other grouse.

## Introduction

Grouse (Galliformes: Tetraoninae) have long been a conservation concern [1]. Many populations are declining and have become threatened, particularly in regions with dense human populations [2]. The capercaillie (*Tetrao urogallus*) is one of the most threatened and best studied species among this group of ground-nesting birds [1]. Conservation approaches designed to boost capercaillie populations include lek site protection [3], mesopredator control [4] and reintroduction of captive-bred chicks [5]. Population decline in the capercaillie and other grouse is often associated with modern forestry practices [2] and in some areas, including Estonia, capercaillie protection is enforced by strict forest management restrictions to drainage and logging in protected areas [6]. Nonetheless, current conservation strategies have not halted capercaillie population declines, with recent evidence suggesting that predators can play a greater role than habitat factors in affecting grouse numbers [7,8].

In Estonia, the overall predation pressure and number of different predators are high in capercaillie protection zones in the early breeding season (May-June, [9]). The main predators of capercaillie appear to be the pine marten (*Martes martes*), raccoon dog (*Nyctereutes procyonoides*), red fox (*Vulpes vulpes*) and goshawk (*Accipiter gentilis*) [8–10], whereas the impact of wild boar (*Sus scrofa*) has been largely neglected and the species is at best considered as an occasional nest predator [11]. The diet of omnivorous wild boar is the function of food availability. Wild boar diet consists primarily of plants, whereas animals are consumed frequently, but mostly in small quantities. Consumed animal food usually consists of carrion, earthworms, insects, small mammals, and reptiles [12]. Although wild boar is also known as a predator of ground-nesting birds and their eggs, particularly grouse [13–18] and waterfowl [19,20], low detection rate of birds in stomachs and faeces explains why wild boar is usually not considered as a conservation concern of ground-nesting birds. During the past decades, wild boar abundance in Europe increased significantly and thus the impact of wild boar on other wildlife is expected to raise [21] with potentially more negative consequences on capercaillie and other ground-nesting birds. However, the majority of wild boar diet studies have relied on morphological examination of consumed food items, a traditional approach to study food habits of mammals [12,22]. Yet, the method has important drawbacks—during the digestion process, consumed food items become highly degraded, making their detection and/or identification dubious. This is also true for consumed bird items, especially when wild boar has eaten eggs or chicks.

We hypothesize that: (a) the proportion of birds in wild boar faeces is underestimated by common morphological methods, and (b) wild boar has significant impact on ground-nesting birds. Our aims were: (1) to develop a non-invasive genetic method to identify both the predator and the bird species by the analysis of mitochondrial DNA isolated from faeces, an approach preferable to stomach contents because non-invasive collection can be carried out also in areas where hunting is prohibited, and 2) to determine the frequency of occurrence of ground-nesting birds, particularly the capercaillie, in wild boar diet. We analysed wild boar faeces collected at capercaillie lek sites during spring-early summer, which is the capercaillie lekking and nesting period, when birds spend a lot of time on the ground and are most vulnerable to predation.

## Materials and methods

### Sample collection

Seven protected capercaillie lekking areas were monitored during three years (2013-2015) from the end of March to the middle of June—the period of capercaillie displaying and breeding in Estonia. Three areas were located in Central Estonia (Lemmjõe, Tipu, and Vanaveski), three in Eastern Estonia (Kaiavere, Muraka, and Sõõru) and one in Southern Estonia (Karula). All of the Central Estonian and one lekking area in Eastern Estonia were situated in a forest-wetland habitat; the rest of the areas were characterised by open coniferous forests. Daily visits to the lekking areas were conducted in order to minimize disturbance to the birds. Wild boar faeces, which are morphologically easily distinguishable from other mammals in Estonia, were systematically collected by searching lekking areas and the surrounding forest. Faeces were put into plastic bags and stored at -80°C before analysis to prevent further degradation of DNA in samples.

### Ethics statement

Samples were collected during the time when movement was restricted in the protected areas of capercaillie. A special permit (No 1–4.1/13/241) was obtained from the Estonian Environmental Board for searching capercaillie lekking areas and the surrounding territories for faeces left by potential predators of capercaillie.

### Molecular analysis

DNA was extracted from 180–220 mg of faeces using QIAamp DNA Stool Mini Kit (Qiagen) according to the manufacturer’s instructions, as described in the protocol “Isolation of DNA from Stool for Human DNA Analysis”. For molecular identification of wild boar scats and prey species, taxon-specific primers (Table 1) were designed. Mammal-specific primer pair Mamm1F/Mamm1R was used to identify faeces belonging to wild boars and bird-specific primer pair Ave12F/Ave12R to identify consumed bird species.

**Table 1:** Primer pairs used to identify mammal and bird species.

Specificity	Name	Primer sequence	Location (mtDNA)	PCR product size (bp)
Mammals	Mamm1F	CAACGGAACAAGTTACCCTAG	16S	188
Mammals	Mamm1R	GAAACCGACCTGGATTACTC	16S	188
Birds	Ave12F	AAGACAGGTCAAGGTATAGC	12S	183
Birds	Ave12R	GAGGGTGACGGGCGGTATG	12S	183

PCR reactions were carried out in a total volume of 20 μl with 1x Phusion HF Buffer (ThermoFisher Scientific, Waltham, US), 0.2 mM dNTP, 0.25 μM of each primer and 0.4U Phusion Hot Start II DNA Polymerase and 2 μl of purified DNA. The PCR mixture was initially denatured at 98°C for 30 s, followed by 10 touchdown cycles for 10 s at 98°C, 20 s at 60°C (reducing the temperature 1°C per cycle) and 30 s at 72°C, followed by 30 cycles of 10 s at 98°C, 20 s at 50°C and 30 s at 72°C.

PCR products were checked using 2% 1xTAE gel-electrophoresis and visualized under UV radiation using ethidium bromide. GeneRuler Ultra Low Range DNA Ladder (ThermoFisher Scientific) was used as a molecular-size marker. In each PCR batch both a negative (reaction mixture without DNA) and a positive control were included to monitor contamination and check for the right amplicons.

PCR products were purified using the Exo-SAP method: to degrade primers and dephosphorylate dNTPs, 1 μl mixture consisting of 1U Exonuclease I (ExoI, 20U/μl) and 1U Alkaline Phosphatase (FastAP, 1U/μl) (ThermoFisher Scientific) was added to 10 μl PCR product. The reaction mixture was incubated for 30 minutes at 37°C, followed by 15 minute enzyme inactivation at 80°C.

DNA-sequencing reactions were conducted for the samples that gave positive results in mammal and/or bird detection, using the same primers as for PCR (Table 1). Sequencing was performed at the Estonian Biocentre core laboratory. Nucleotide BLAST (http://blast.ncbi.nlm.nih.gov/) was used to identify mammal and bird taxa.

### Morphological analysis

Faeces were washed on a metal sieve (0.8 mm mesh size) and identifiable objects were divided into eight categories: greens (above-ground plant material, except for tree leaves and needles), roots, supplementary (grain from feeding sites), invertebrates (earthworm, arthropod wings and leg segments, fragments of chitin), mammals (hair, teeth and bone fragments), birds (feathers, bone and eggshell fragments), birds/reptiles (scales crushed into fragments too small for certain identification), other (digested fragments of tree leaves and needles, small stones, etc.).

To quantify diet composition, frequency of occurrence (FO = number of faeces containing each food category / total number of faeces) and volume of each food category were measured. Items were placed into a measuring cylinder by category, and the volume (ml) of each category was measured by water displacement (volumetric evaluation). Owing to the difference in faeces size, relative volume percentage was calculated, using the formula $V_{a}\%=100\times \frac{\sum V_{a}}{V}$, where *V*_*a*_% is the percentage volume of a food category, ∑*V*_*a*_ is the volume of objects in a category, and *V* is the total volume of objects in all categories.

## Results

**Fig 1:**
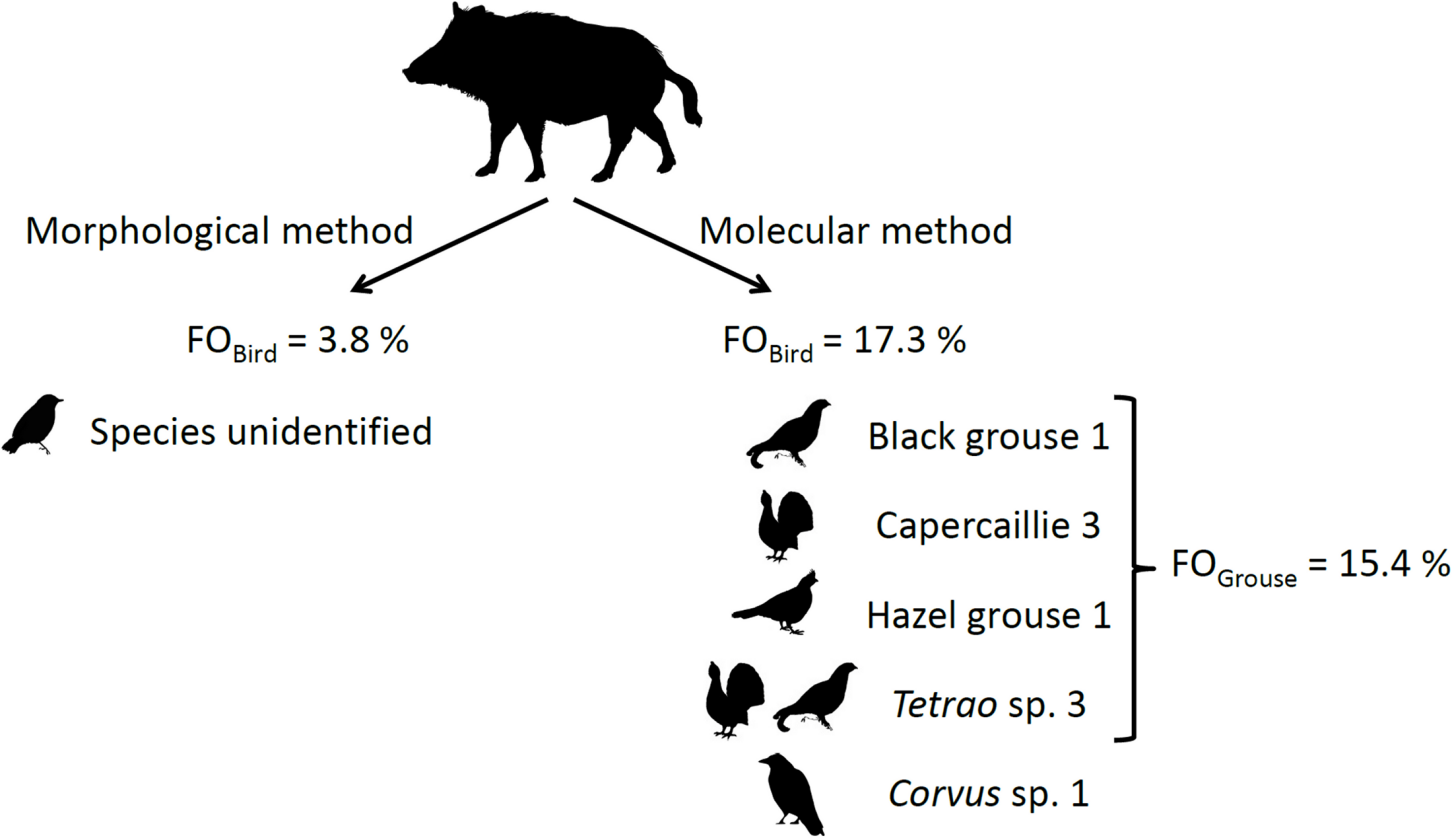
Bird consumption by wild boar in Estonia: Molecular method 4.5× more sensitive than the morphological. FO_Bird_−frequency of occurrence of bird in wild boar diet; FO_Grouse_−frequency of occurrence of grouse in wild boar diet.

Of 109 wild boar faeces analysed with the molecular method, 49 were identified as wild boar and of these in 6 we identified bird DNA. Moreover, among the samples that gave negative result in mammalian PCR, the genetic method identified bird DNA in three samples. Although the genetic analysis could not identify the predator for three faecal samples, either because the sample had degraded or included inhibitors that prevented DNA amplification, they clearly belonged to wild boar (based on morphology). Thus, including the three samples in which the genetic analysis failed to identify the mammalian predator, in total we identified 9 faeces with bird DNA from 52 wild boar samples (FO = 17.3%, Fig 1). Of these, 5 were determined to species level (9.6%): 3 capercaillie, 1 black grouse (*Tetrao tetrix*) and 1 hazel grouse (*Tetrastes bonasia*). Due to partial sequences, the other four bird-positive samples were determined to genus level: three to *Tetrao* and one to *Corvus*. In comparison, the morphological analysis of wild boar faeces (N = 52) identified only two cases of bird consumption (FO = 3.8%), containing both feathers and bone fragments of adult birds, and failed to detect birds in six samples where bird consumption was confirmed with genetic analysis. In two cases the molecular method helped to specify morphological category bird/reptile, one of which turned out to be black grouse. Thus, the sensitivity of genetic analysis is over 4.5 times higher compared to the morphological approach (Fig 1). The majority of wild boar diet consisted of plants (FO = 78.9%) and the presence of supplementary food originating from the surrounding hunting areas was also relatively high (FO = 30.8%). However, six of the collected faeces (5.5%) contained no plant material: two contained bird and invertebrates; one indicated nest predation and mammal predation (field vole *Microtus agrestis*, identified by teeth morphology); and the remaining three consisted of bird/reptile, carrion (roe deer *Capreolus capreolus*, identified by hair morphology) or invertebrate.

Morphological analysis of the full dataset (N = 109; S1 Table) revealed two further samples indicating bird predation. However, feathers and fragments of egg shells were too degraded for morphological identification of bird taxa in these samples. In addition, prey items belonging to bird/reptile were identified and in two such cases the molecular method helped to specify that one of the sequences belonged to black grouse, whereas the other to a reptile from the order Squamata.

## Discussion

Non-invasive sampling coupled with molecular identification continues to be an inestimable method in conservation biology, ecology and various other biological research areas [23–25]. The benefit of using this approach is at least two-fold. Firstly, the method allows sampling without disturbing animals, and secondly, it provides means for accurate identification of animal species who has left the faeces (or feather, hair, saliva, urine, etc.), which can be used as a source of DNA. Non-invasive genetics has been a fruitful approach for analysing food habits, parasites and spatial movements of various animal taxa [26–29]. For this study we designed universal primers for mammals and birds, which are not restricted to identify only ungulates or grouse, but can be applied for the genetic identification of a wide number of mammal and bird species. Here we chose the traditional Sanger sequencing instead of metabarcoding. The latter approach is sometimes a valuable alternative to the traditional Sanger sequencing, especially if the aim is simultaneous detection of multiple taxonomic groups of organisms (e.g. plants, invertebrates) from complex diet samples [30]. However, for this study the Sanger sequencing was an optimal solution since it is unlikely that wild boars consume more than one bird species during a meal. The number of ground-nesting birds is rather low in our study area and bird consumption by wild boar is known to be a rare event [12,13,20]. Therefore, the traditional sequencing is sufficient to reveal the taxonomic identity of the bird, and the predator. Moreover, it is also cheaper and easier to apply.

Although we could genetically identify 49 samples as wild boars, morphologically all collected faeces clearly belonged to this species. While animal identification based on scat morphology is often difficult for meso-predator species [31], wild boar faeces are distinct from any other mammal. Nonetheless, we applied the genetic method for predator identification to confirm that genetic analysis is able to identify wild boars with confidence. However, detecting and identifying bird remains in wild boar faeces using morphological approach is problematic due to high level of degradation. Indeed, comparison of genetic and morphological analysis (N = 52) in detecting birds in wild boar diet revealed that genetic approach was considerably (4.5×) more sensitive. Although morphological analysis can be used to obtain additional information for prey (e.g. age class of birds), its sensitivity is inferior to the molecular method. Thus, molecular analysis is a powerful tool for identifying food items that remain morphologically undetected or unidentifiable. We suggest combining genetic and morphological approaches to extract maximum amount of dietary information from faeces. This is probably particularly important for detecting young chicks and cases of nest predation in faecal analysis.

In addition to grouse, a variety of passerines, pigeons, woodpeckers, owls and waterfowl have been identified in previous studies in wild boar diet [13,20,32–34]. However, our results show that wild boars predated almost exclusively on ground-nesting grouse, whereas capercaillie was targeted more often than other grouse species present in the area (see also [11]). Wild boars often use coniferous forests, which are typically scarce in natural food, for day-time resting [35]. The probability of preying on vertebrates is negatively related to wild boar body condition [34] and, therefore, bird consumption in these areas may be vital to fulfil the energy requirements [12]. Also, during the first few weeks after hatching, when chick mortality is highest, female capercaillie with broods select habitats that are rich in invertebrates [36], attractive also to wild boars.

Despite its low volume, animal food is arguably an essential dietary component for wild boar, reaching up to 88% of food intake [37], and can be obtained by predation and scavenging [12]. We suggest that in most cases wild boar acted as a predator. Firstly, faeces were collected in spring and early summer when eggs (containing foetuses) and chicks are readily available, though adults can be also killed or consumed as carrion. Consumption of adult birds leaves identifiable signs in faeces such as feathers and bone fragments, whereas foetuses and freshly hatched chicks can be entirely digested and detected only in genetic analysis. However, in majority of cases (seven out of nine) we could detect bird only in genetic analysis, suggesting predation of foetuses or chicks. Secondly, as mesopredators in our study area also feed on carrion, all carrion is consumed quickly and it is highly unlikely that wild boars had an opportunity to eat carrion in all the registered cases. Thirdly, we found six samples that consisted entirely of animal matter and contained no plant material, whereas five of these contained small mammal, bird and/or invertebrate, and only one carrion (roe deer). Therefore, our results suggest deliberate consumption of animal food as opposed to the opportunistic predation while rooting, indicating that wild boar can also act as a predator and seek out its prey. Similarly, Wilcox and van Vuren [34] found that predation is not an occasional event and wild boar searches for prey, including agile terrestrial vertebrates. Such behaviour would require regular visits to areas with an increased probability of catching the prey. Indeed, one of the faeces containing adult bird feathers was collected near a predated nest, suggesting repeated use of grouse habitat by the same boar.

In addition to natural food items, supplementary food (grain) was also frequently detected in wild boar faeces (see S1 Table). Capercaillie protection zones (ranging 1 km around leks) in Estonia have restrictions against supplementary feeding of wild boar (since 2007) and other game species (since 2013) as a means to prevent attracting potential predators to capercaillie areas. However, game management in hunting districts surrounding the protection zones are currently unregulated with regards to supplementary feeding. Such regulations have created a matrix for dissuasive feeding, which can sometimes prevent damage to farmland [38] and has been recommended as a measure to prevent ground nest predation [39]. However, supplementary feeding is known to produce various unintended effects [40] and in Estonia, high densities of wild boar [41] and other generalist mammals (e.g. raccoon dog [42]) have been sustained by supplementary feeding. It is clear that the availability of supplementary food did not prevent wild boar from preying on grouse. Wild boar depends on agricultural crops [12], also in the form of supplementary food [33], and their home ranges and daily movements exceed the size of protection zones targeted in this study [43]. The spatial scale of feeding restrictions (1 km) therefore appears insufficient to keep the wild boar away from protected areas.

We conclude that for efficient conservation of ground-nesting birds, the wild boar should be considered as an important predator of capercaillie and other grouse, especially in areas where wild boar densities are high. Compared to the traditional morphological analysis, non-invasive genetics proved to be considerably more efficient in identifying birds consumed by wild boars. This suggests that many previous studies based on morphological methods may have underestimated the rate of bird consumption by omnivorous mammalian predators and can have important consequences on conservation decisions that depend on the results of dietary studies. Based on the results of this study, we recommend using genetic methods in all trophic studies where the food category of interest is not easily detectable or identifiable due to high level of degradation.

## Supporting information

S1 Table Morphological analysis of food items in wild boar faeces. FO % – percentage of frequency of occurrence; Volume % – percentage volume of different food categories. The smaller dataset (N = 52) includes wild boar faeces that gave a positive result with the molecular analysis, the larger dataset (N = 109) includes all samples that were collected and morphologically identified as belonging to wild boar.
(PDF)
