# Supplementary material for: Non-invasive genetics outperforms morphological methods in faecal dietary analysis, revealing wild boar as a considerable conservation concern for ground-nesting birds
Source: PLoS One. 2017 Jun 8;12(6):e0179463. doi: 10.1371/journal.pone.0179463 (PMC5464655; doi:10.1371/journal.pone.0179463)
Supplement: S1 Table — FO % – percentage of frequency of occurrence; Volume % – percentage volume of different food categories. The smaller dataset (N = 52) includes wild boar faeces that gave a positive result with the molecular analysis, the larger dataset (N = 109) includes all samples that were collected and morphologically identified as belonging to wild boar. (PDF) [file pone.0179463.s001.pdf]

Non-invasive genetics outperforms morphological methods in faecal dietary analysis, revealing wild boar as a considerable conservation concern for ground-nesting birds

Ragne Oja[1], Egle Soe[1], Harri Valdmann[1], Urmas Saarma[1,*]

[1] Department of Zoology, Institute of Ecology and Earth Sciences, University of Tartu, Tartu, Estonia

* Urmas.Saarma@ut.ee

S1 Table. Morphological analysis of food items in wild boar faeces.

	Wild boar faeces (N = 52)		Wild boar faeces (N = 109)	
**Category**	**FO (%)**	**Volume (%)**	**FO (%)**	**Volume (%)**
Greens	23.1	15.6	46.8	38.2
Roots	55.8	35.7	37.6	23.6
Supplementary	30.8	25.5	25.7	21.2
Invertebrates	19.2	2.7	25.7	3.5
Mammals	5.8	1.0	7.3	1.9
Birds	3.8	1.5	3.7	1.2
Birds/Reptiles	34.6	15.6	17.4	7.4
Other	25.0	3.4	21.1	3.0

FO % – percentage of frequency of occurrence; Volume % – percentage volume of different food categories. The smaller dataset (N = 52) includes wild boar faeces that gave a positive result with the molecular analysis, the larger dataset (N = 109) includes all samples that were collected and morphologically identified as belonging to wild boar.
